# Hexokinases and Glial Tumor Metabolism: A Bridge between Bioenergetics and Translational Oncology

**DOI:** 10.32604/or.2026.079467

**Published:** 2026-10-28

**Authors:** Corina Ionela Tamas, Flaviu Tamas, Alina Roxana Cehan, Adrian Balasa

**Affiliations:** 1 Department of Neurosurgery, “George Emil Palade” University of Medicine, Pharmacy, Science and Technology, Targu Mures, Romania; 2 Department of Neurosurgery, Emergency Clinical County Hospital, Targu Mures, Romania; 3 Doctoral School of Medicine and Pharmacy, “George Emil Palade” University of Medicine, Pharmacy, Science and Technology, Targu Mures, Romania

**Keywords:** Hexokinases, glial tumors, metabolism

## Abstract

Hexokinases, particularly hexokinase 2, play a central role in the metabolic reprogramming of glioblastoma and other malignant glial tumors by promoting aerobic glycolysis and sustaining the Warburg phenotype. This review aims to summarize the current evidence regarding the molecular regulation, biological significance, and therapeutic implications of hexokinase isoenzymes in glial tumor metabolism. Recent studies consistently demonstrate that hexokinase 2 overexpression is associated with enhanced tumor proliferation, invasiveness, angiogenesis, resistance to chemotherapy and radiotherapy, and poor prognosis, especially in glioblastoma and high-grade gliomas. Multiple regulatory mechanisms, including microRNAs, long non-coding RNAs, the phosphatidylinositol 3-kinase/protein kinase B signaling pathway, and hypoxia-inducible factor 1-alpha-mediated signaling, as well as mitochondrial interactions through the voltage-dependent anion channel, contribute to metabolic adaptation and tumor progression. In addition to its metabolic role, hexokinase 2 has emerged as an important mediator of the immunosuppressive tumor microenvironment and maintenance of the malignant phenotype. Overall, targeting hexokinase 2 and related glycolytic pathways represents a promising strategy for personalized metabolic therapy and may improve responsiveness to conventional oncologic treatments in patients with gliomas.

## 1 Introduction

The proliferation of tumor cells requires the presence of a metabolic microenvironment that supports their growth and survival [1]. This perspective has contributed to a deeper understanding of the fundamental mechanisms underlying tumor behavior and has enabled the identification of specific oncometabolites associated with various neoplastic entities, such as 2-hydroxyglutarate (D2-HG), which are currently used in diagnosis and in assessing tumor aggressiveness [2,3].

In astrocytic tumors, metabolic reprogramming plays a central role in sustaining growth and progression. Increased glucose consumption, lactate accumulation through the Warburg effect, and glutamine dependence provide the bioenergetic and biosynthetic requirements for the production of essential macromolecules (lipids, carbohydrates, proteins, nucleic acids), while maintaining the redox balance necessary for cellular proliferation [4]. Furthermore, metabolites generated through these pathways can act as signaling molecules, amplifying oncogenic processes. A detailed understanding of the tumor metabolic profile is therefore a key premise for the development of novel therapeutic strategies in gliomas [5].

Glycolysis, as an essential metabolic pathway, provides not only a rapid source of energy but also intermediates required for macromolecular biosynthesis, which supports cell proliferation and tumor progression [6]. The process takes place in the cytosol, under both aerobic and anaerobic conditions, and involves the oxidation of one molecule of glucose to produce two molecules of adenosine triphosphate (ATP) and two molecules of nicotinamide adenine dinucleotide in its reduced form (NADH), along with two molecules of pyruvate [7,8]. In the presence of oxygen, pyruvate is directed into the mitochondria for oxidative phosphorylation (OXPHOS), whereas in its absence, it remains in the cytosol and is converted to lactate by the action of lactate dehydrogenase (LDH) [9].

In normal cells, pyruvate generated from glycolysis is completely oxidized through the tricarboxylic acid (TCA) cycle and OXPHOS, yielding up to 36 molecules of ATP per glucose molecule [10]. In contrast, tumor cells preferentially engage in aerobic glycolysis, converting most of the glucose to lactate regardless of oxygen availability (the Warburg effect). This strategy allows the redirection of metabolites toward anabolic pathways that support rapid proliferation at the expense of energetic efficiency [11].

Beyond its energetic and biosynthetic roles, glycolysis contributes to the remodeling of the tumor microenvironment and influences immune responses [12,13]. It sustains critical processes such as tumor growth, invasion, angiogenesis, and resistance to standard oncologic therapies. Recent studies suggest that shifting tumor metabolism from glycolysis toward OXPHOS may promote the differentiation of glioblastoma (GBM) cells into astrocytes, thereby reducing tumor aggressiveness [14]. In this context, rate-limiting glycolytic enzymes—particularly hexokinase 2 (HK2) and pyruvate kinase M2 (PKM2)—have been associated with tumor development and resistance to apoptosis. Selective inhibition of these enzymes represents a promising therapeutic strategy with the potential to directly target tumor cells and reduce their survival [15,16,17].

The aim of this review is to provide an in-depth analysis of the role of HKs, particularly HK2, as key enzymes in the metabolic reprogramming of gliomas, emphasizing their correlations with molecular markers, malignant phenotype, and potential therapeutic applications in the context of modern metabolic oncology.

## 2 Hexokinase—Functions and Isoforms

### 2.1 Role and Mechanism of the Hexokinase-Catalyzed Reaction

HKs are essential enzymes in the initiation of glycolysis, catalyzing the phosphorylation of glucose and related hexoses at the sixth carbon position. In this reaction, the terminal phosphate group of ATP is transferred to the oxygen bound to carbon 6 of the glucose molecule, generating glucose-6-phosphate (G6P) and ADP [18]. The effective phosphate donor is the ATP–Mg^2^^+^ complex, and the resulting reaction is strongly exergonic (ΔG° ≈ −16.7 kJ/mol), making it practically irreversible under physiological conditions (ΔG < −30 kJ/mol) [19,20].

From a structural perspective, HKs are classified into four major isoforms—HK I, II, III, and IV (also known as A, B, C, and D)—each encoded by a distinct gene and adapted for specific metabolic functions in different cell types [19]. A fifth form, hexokinase domain-containing 1 (HKDC1), has been recently identified at the genomic level, though its functional role remains poorly characterized. HK I–III are monomeric proteins of approximately 100 kDa, believed to have originated from the duplication of an ancestral gene encoding a 50 kDa hexokinase. These isoforms exhibit very low Michaelis–Menten constants (Km) for glucose (<0.2 mM), indicating a high substrate affinity, and are inhibited by physiological concentrations of G6P [21,22]. Among them, HK III displays an extremely low Km for glucose, though its activity decreases at high substrate concentrations. HK IV (glucokinase, GK) is a distinct 50 kDa protein with a low affinity for glucose and sigmoidal kinetics. Unlike the other isoforms, it is not inhibited by G6P but is regulated by a specific protein, the glucokinase regulatory protein (GKRP) [21,22]. Although it can phosphorylate other hexoses, its physiological specificity is almost exclusively directed toward glucose—the predominant intracellular substrate. Owing to this property, GK is considered a true “glucose sensor”, playing a critical role in maintaining energy homeostasis. The Km value remains a fundamental indicator of an enzyme’s affinity for its substrate: a low constant reflects high affinity, suggesting efficient catalytic activity even at low glucose concentrations. This relationship defines the distinct behavior of HK isoforms across various tissues and pathophysiological contexts, including in neoplastic cells, where their regulation acquires metabolic and therapeutic significance (Table 1, [19]) [23,24].

**Table 1:** Kinetic and Regulatory Characteristics of Human HK Isoforms. Although these isoforms share structural and functional similarities due to a common evolutionary origin, they exhibit significant differences in kinetic properties, regulatory mechanisms, intracellular localization, and tissue expression patterns.

Characteristic	HK1	HK2	HK3	HK4 (GK)
Gene locus	10q22	2p13	5q35.2	7p15.1–3
Molecular weight (kDa)	~100	~100	~100	~50
Km for glucose (mM)	0.03	0.3	0.003^a^	6
Km for ATP (mM)	0.5	0.7	1	0.6
G6P inhibition (Ki, mM)	+ (0.02)	+ (0.02)	+ (0.1)	x
Pi attenuation of G6P inhibition	↓	↑	↓	x
Insulin regulation	x	+	?	+
Mitochondrial binding	+	+	x	x
Main tissue expression	Brain, kidney	Muscle, adipose tissue	Lung, spleen	Liver, pancreas

Note: +, present; ↑ = increase; ↓ = decrease; ×, absent; ?, unknown; ^a^, substrate inhibition of HK3 activity observed *in vitro* at glucose concentrations > 1 mM; Km, Michaelis–Menten constant; Ki, inhibition constant; Pi, inorganic phosphate.

### 2.2 Hexokinase Isoforms: Functional Characteristics and Oncological Relevance. Differential Isoform Expression and Mitochondrial Interaction

The diversity of kinetic parameters highlights the complex regulatory network governing HKs’ activity according to cellular energy demands and tissue type (Table 1) [19]. In cells that rely on a constant glucose supply, such as neurons and erythrocytes, the activity of HK I and HK III is continuously maintained due to their high substrate affinity and strict feedback inhibition by G6P. In contrast, HK II exhibits a dual specificity: a moderate affinity for glucose and a predominant mitochondrial localization, which allows it to directly link glucose utilization with ATP production and macromolecular biosynthesis—processes essential for proliferative and tumor cells. HK IV displays distinct behavior, being insensitive to G6P inhibition and activated at high glucose concentrations [18,19]. These properties make it a finely tuned metabolic sensor, perfectly adapted to its hepatic and pancreatic role in maintaining glucose homeostasis. Overall, variations in Km and Ki values reflect not only the differential substrate affinity but also the subtle mechanisms that control glycolytic flux, ensuring a balance between cellular energy requirements and the maintenance of systemic glucose levels [25,26].

The ubiquitous isoform HK I is constitutively expressed in most tissues, ensuring a constant glucose utilization in cells with high energy demands, such as neurons and erythrocytes. HK II, the main regulatory isoform, predominates in insulin-sensitive tissues—skeletal muscle and adipose tissue—where it accounts for the majority of peripheral glucose consumption [19]. This isoform is also highly expressed in many types of cancer characterized by enhanced glycolytic activity, linking it directly to the Warburg effect. Although HK III is present across a wide range of tissues, it is not the dominant isoform in any specific cellular compartment, and the mechanisms regulating its expression remain poorly understood. Differences in sensitivity to feedback inhibition by G6P distinguish these high-affinity isoforms [22,27]. HK I and HK II are strongly inhibited by G6P, a process finely modulated by inorganic phosphate (Pi), which mitigates the inhibitory effect. Conversely, Pi directly inhibits HK II and HK III, adding an additional layer of control over glycolytic flux. HK III exhibits a higher affinity for glucose and lower sensitivity to G6P inhibition compared to HK I and HK II, while also being subject to substrate inhibition at physiological glucose concentrations—a phenomenon partially counteracted by intracellular ATP levels [28]. The absence of mitochondrial localization and its distinct kinetic profile suggest that HK III may broaden the glucose phosphorylation capacity in cells co-expressing multiple isoforms, although the evolutionary rationale for this remains uncertain [29].

HK regulation is a complex process that involves not only differential gene expression but also post-translational modifications, intracellular compartmentalization, substrate availability, and allosteric interactions [30]. Among all isoforms, HK I and HK II possess a unique characteristic: a physical and functional interaction with mitochondria. These enzymes bind to the outer mitochondrial membrane via the voltage-dependent anion channel (VDAC)—an association that enhances catalytic efficiency and shields the enzymes from G6P-mediated inhibition [31]. The binding of HK to mitochondria is reversible and modulated by various factors, including ATP, G6P, divalent cations, and intracellular pH. This coupling enables the direct use of mitochondrial ATP for glucose phosphorylation, maintaining glycolytic flux even under metabolic stress. Moreover, mitochondrial HK activity is frequently upregulated in tumor cells, where it supports neoplastic growth and survival [32].

### 2.3 Structural Features and the Role of HKs in Brain Metabolism

Structurally, the C-terminal domain of HK I is responsible for catalytic activity, while the N-terminal domain, which is enzymatically inactive, modulates the enzyme’s sensitivity to Pi and G6P [33,34]. In contrast, HK II exhibits comparable catalytic activity in both domains. The defining feature of HK I, which predominates in brain tissue, is its ability to have G6P-induced inhibition reversed by physiological concentrations of Pi, granting it a unique role in regulating cerebral glycolytic flux [33,35]. These differences in expression, localization, and post-translational regulation highlight the fine adaptation of each HK isoform to the specific energetic and metabolic requirements of distinct cell types, reflecting an evolutionary balance between efficiency, control, and metabolic flexibility (Fig. 1) [33,36].

**Figure 1:**
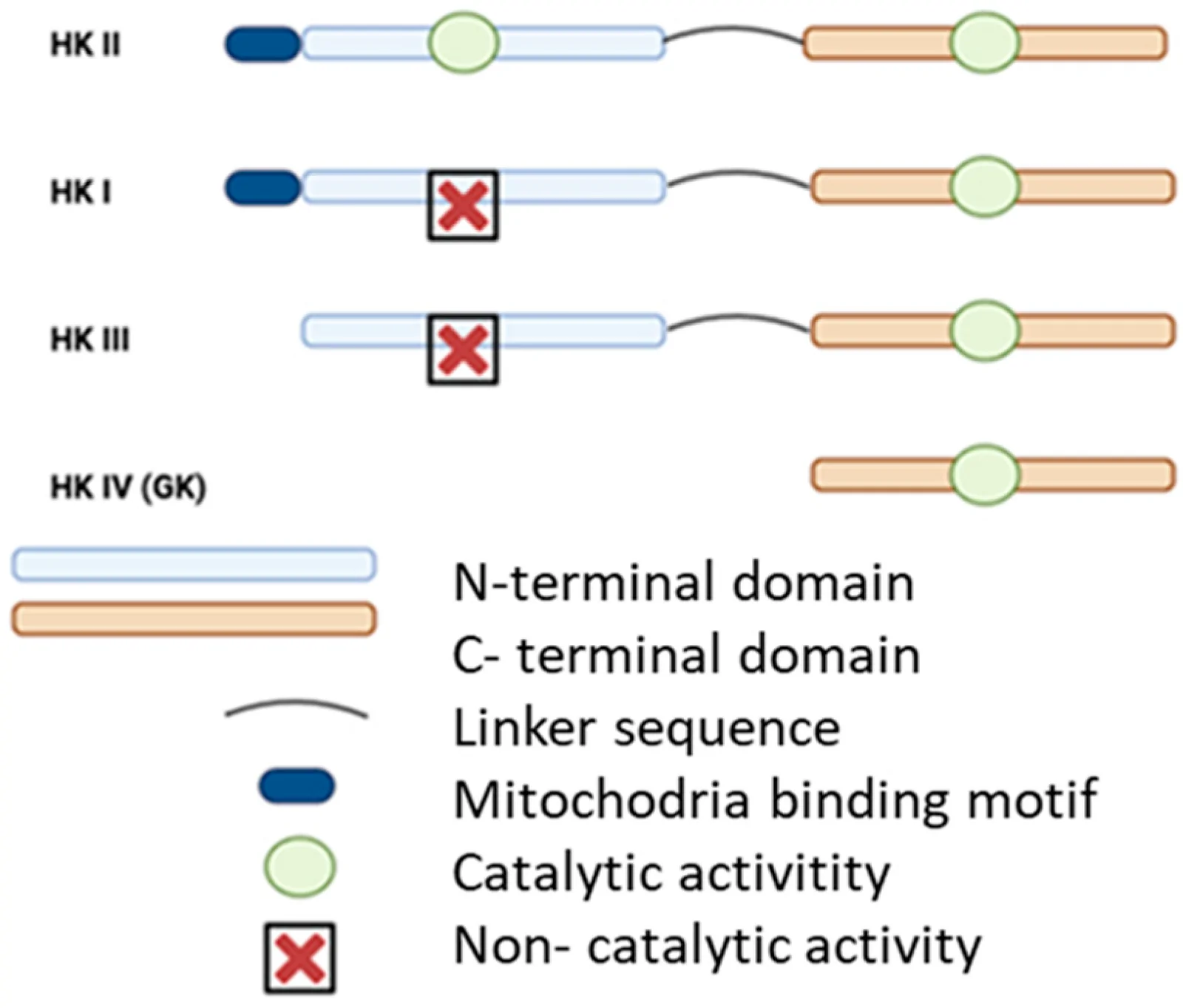
Structure of HKs and mitochondrial-binding domain. Comparison of HK I, HK II, HK III, and HK IV structures. HK II possesses two active catalytic domains and a mitochondrial-binding motif at the N-terminus. HK I has catalytic activity only in the C-terminal domain. HK III exhibits a similar structure to HK I. HK IV (glucokinase) lacks a mitochondrial-binding motif and is specific to the liver and pancreas. Created with BioRender.com (BioRender Inc., version 2.1, 2022, https://www.biorender.com).

Approximately 40% of total circulating glucose is used to sustain the brain’s energy metabolism [33]. Over a single day, the brain consumes an amount of glucose equivalent to about 13% of its own weight. The conversion of glucose to G6P, mediated by the HK I isoform, keeps glucose in a phosphorylated form that cannot leave the cell, thus committing it to intracellular energy-generating pathways [33,37]. Moreover, HK I has a Km for glucose nearly 100 times lower than the normal circulating glucose concentration (5 mM), ensuring that even under starvation conditions, the brain can maintain active glycolysis and adequate energy production. However, the continuous activity of an enzyme that sustains glycolysis even during fasting can lead to metabolic imbalances when glucose availability is sufficient [33,38]. If the entire intracellular glucose pool were converted to G6P, the resulting depletion of Pi could fall to levels insufficient to support OXPHOS. To prevent this, HK I is tightly regulated by both G6P and Pi concentrations. Thus, the brain’s high glucose demand is met through precise regulation at the first step of glycolysis, where the interplay between the N-terminal (regulatory, non-catalytic) and C-terminal (catalytic) domains serves as the main control mechanism of HK I (Fig. 1) [33,39].

## 3 Metabolic Mechanisms of Hexokinases in Glioma

The upregulation of HK II has been identified as a major factor that enhances glycolysis, even in the presence of oxygen, exhibiting the Warburg effect. During tumor development in tissues that normally express HK IV, HK II expression is induced while HK IV is suppressed [40]. This switch from a lower-affinity isoform to a higher-affinity one may provide the energetic support required for tumor proliferation. HK II expression levels are closely correlated with tumor grade and mortality in hepatocellular carcinoma, Table 2. In normal brain tissue and low-grade glioma (LGG), HK I remains the predominant isoform; however, in GBM, HK II is upregulated, and poor prognosis is associated with HK II upregulation, as also observed in cerebral metastases of breast cancer. This upregulation is considered a consequence of metabolic reprogramming in cancer. Beyond its fundamental metabolic role, HK II acts as a protective molecule, exerting antioxidant effects, safeguarding mitochondria against stress, and facilitating autophagy under starvation conditions. Direct interactions of HK II with various molecular pathways, such as the Akt/mTOR pathway, allow the integration of metabolism with cell survival and provide adaptive mechanisms in response to changes in the cellular environment [41].

Importantly, the role of hexokinase isoforms differs across distinct glial tumor entities. In LGGs and mixed-grade glioma cohorts, HK1 remains the predominant isoform, while HK2 expression progressively increases with tumor grade, reflecting a gradual shift toward a more glycolytic phenotype. Studies including WHO grade II–IV gliomas suggest that metabolic reprogramming begins early during glioma progression and intensifies as tumors evolve toward higher-grade lesions [42,43,44]. This distinction between LGG and GBM is essential for the interpretation of the studies summarized in Table 2, as the biological significance of HK2 expression differs substantially according to tumor grade and molecular subtype. Wolf et al. demonstrated that in GBM, HK2 is a central mediator of aerobic glycolysis and tumor growth, suggesting that in grade IV tumors a consolidated metabolic dependency emerges [45]. In a complementary manner, Kim et al. and Du et al. showed that during the transition from grade II–III gliomas to GBM, HK2 is finely regulated through epigenetic and post-transcriptional mechanisms (miR-542-3p and miR-9), indicating that lower-grade tumors maintain greater metabolic plasticity and an incomplete activation of the Warburg axis [21,43]. Furthermore, Park et al. demonstrated that in GBM, NOX2 activation amplifies HK2 expression and directly links glycolytic metabolism to the mesenchymal phenotype and tumor aggressiveness, suggesting that progression to grade IV involves not only increased metabolic flux but also its coupling with invasive remodeling [44]. At the same time, Chen et al. add an additional layer of complexity by showing that HLA-F stabilizes HK2 at the protein level, thereby reinforcing a metabolically stable phenotype that is less dependent on the transcriptional regulatory mechanisms observed in LGGs [46]. Thus, integrating these findings within the molecular heterogeneity of glioma, it becomes evident that progression from grade II–III to GBM is characterized by a shift from partially reprogrammed metabolism—shaped by epigenetic factors and IDH1 status—to a fixed metabolic phenotype driven by the convergent activation of multiple upstream pathways that stabilize HK2 as a key node of tumor aggressiveness.

Several studies suggest that HK 2 overexpression is not merely a consequence of metabolic reprogramming but an active element in tumor progression, involved in regulating complex molecular axes that include microRNAs, long non-coding RNAs (lncRNAs), and transcription factors. These interactions influence tumor phenotype, response to chemotherapeutic treatments, and the transition to more aggressive cellular states, such as the mesenchymal phenotype. Table 2 summarizes key studies on HK2 in glioma, detailing the investigated mechanisms or targets, observed effects on tumors, therapeutic potential, and suggested directions for future research.

**Table 2:** The mechanisms by which HKs modulate tumor metabolism in glioma.

Study	Glial Entity/Tumor Type	Model	Mechanism/Target	Effect on Tumor	Therapeutic Potential	Future Directions
**Zhao et al.** [47] ***In vitro*/Cell lines**	GBM/glioblastoma stem-like cells (GSLCs)	Human GBM tissues, U87, C6, GBM-1 GSLCs, *in vivo* xenograft	miR-143 → HK2	↓ glycolysis, ↓ stemness, ↓ proliferation, ↑ differentiation	miR-143 restoration; HK2 inhibition; combination with 2-DG	Explore miR-143-based metabolic therapy in GBM
**Wolf et al.** [45] ***In vitro*/*In vivo* (GBM models)**	GBM	*In vitro* cell lines + *in vivo* xenograft models	HK2 → Warburg effect	↑ glycolysis, tumor growth, therapy resistance	HK2 inhibition may enhance chemo/radiotherapy sensitivity	Develop HK2 inhibitors; modulate astrocytic tumor metabolism
**Zhang et al.** [42] ***In vitro*/*In vivo* (mouse xenograft)**	Glioma (WHO grades I–IV)	Human glioma tissue samples + *in vitro* glioma cell lines (U251, T98G, A172, LN229, HS683)	ZFAS1 → miR-1271-5p → HK2	↑ proliferation & migration, ↓ apoptosis; tumor growth *in vivo*	ZFAS1 inhibition; miR-1271-5p overexpression	Investigate molecular mechanisms and therapeutic potential
**Zhang et al.** [48] ***In vitro*/*In vivo***	GBM	Human GBM tissues + cell lines + *in vivo* xenograft models	HOTAIR → miR-125 → HK2	↑ proliferation, ↑ TMZ resistance, ↓ apoptosis, tumor progression	Target HOTAIR/miR-125/HK2 axis to restore TMZ sensitivity	Develop RNA-based therapies targeting chemoresistance pathways
**Dong et al.** [49] ***In vitro***	GBM, IDH1 wild-type	IDH1WT GBM cell lines (*in vitro*)	snoRNA U3 → ZBTB7A → HK2/LDHA	↑ glycolysis & proliferation in IDH1-wt GBM	Target U3/ZBTB7A/HK2–LDHA axis to inhibit glycolysis	Explore inhibitors and other snoRNA effects
**Huang et al.** [50] ***In vitro*/Tissue analysis**	Non-glial cancer models	HepG2 + HeLa + HEK293T + CD34^+^ cells	m6A/IGF2BP1-3 → oncogenic mRNA (MYC) stabilization	↑ proliferation & survival	IGF2BP–m6A inhibition	Explore IGF2BP inhibitors
**Du et al.** [43] ***In vitro***	Glioma (WHO grades II–IV)	Human glioma tissue samples + PTBE controls + *in vitro* glioma cell lines (LN229, SHG44, T98G, U251-MG)	ANXA2P2 → miR-9 → LDHA	↑ glycolysis & proliferation	ANXA2P2 inhibition; miR-9 upregulation	Reduce glycolytic activity and tumor growth
**Krajewska et al.** [51] ***In vitro*/Molecular docking**	GBM	U-87, U-251 cell lines + HKII docking/NMR model	Halogenated 2-DG analogs → HK2	↓ glycolysis, ↓ lactate synthesis, ↓ proliferation, ↑cytotoxic under hypoxia	2-DG analogs as metabolic therapy	Test *in vivo* and combination therapies
**Kim et al.** [21] ***In vitro*/Tissue Correlation**	Human glioma tissues (grade I–IV)	U87MG cell-line model, TCGA LGG/GBM cohort (*n* = 689)	miR-542-3p → HK2	↑ glycolysis & proliferation, ↑ glycolytic flux (ECAR), poor survival	miR-542-3p inhibition	Explore miR-542-3p inhibition as therapy
**Park et al.** [44] ***In vitro*/Tissue analysis**	GBM/Glioma (mixed grades I–IV; human tissue + GBM cell model)	Human tissue cohorts + U87MG cells	NOX2 → HK2 → mesenchymal transition (COL5A1, FN1)	↑ glycolysis, mesenchymal phenotype, ↑ ECM remodeling	NOX2 inhibition	Evaluate NOX2 inhibition strategies
**Liu et al.** [52] ***In vitro*/*In vivo*/** **Metabolomics**	GBM/HGG	Orthotopic (U87) and subcutaneous xenograft mouse models; human glioma cell lines; RNA-seq; WGCNA; ChIP-qPCR; co-IP; ubiquitination assays; Seahorse metabolic flux; LC–MS metabolomics	RCC2 → BACH1 → HK2; RCC2 → G2/M cell cycle	↑ proliferation, ↑ migration, ↑ glycolysis, ↑ metabolic adaptation and tumor growth	Target RCC2 to limit tumor growth & metabolic adaptation	Investigate RCC2 as a therapeutic target
**Chen et al.** [46] ***In vitro*/*In vivo***	Glioma (multiple grades: astrocytoma grade I–III; glioma cell lines)	Human glioma cell lines (U87, U251, T98G, etc.), murine astrocytes (C8-D1A), xenograft models in Rag2−/− mice, human tissue microarray	HLA-F → HK2	↑ glycolysis, ↑ proliferation, ↑ tumor recurrence, ↑ tumor growth	HLA-F inhibition alongside HK2 targeting	Develop strategies targeting HLA-F and HK2

Note: ↑ = increase;↓ = decrease; GBM, glioblastoma; GSLCs, glioblastoma stem-like cells; WHO, World Health Organization; IDH1WT, isocitrate dehydrogenase 1 wild-type; PTBE, peritumoral brain edema; TMZ, temozolomide; ECM, extracellular matrix; LGG, low-grade glioma; HGG, high grade glioma, TCGA, The Cancer Genome Atlas; RNA-seq, RNA sequencing; WGCNA, weighted gene co-expression network analysis; ChIP-qPCR, chromatin immunoprecipitation quantitative polymerase chain reaction; co-IP, co-immunoprecipitation; LC–MS, liquid chromatography–mass spectrometry; NMR, nuclear magnetic resonance; ECAR, extracellular acidification rate; 2-DG, 2-deoxy-D-glucose.

Among the tumor entities summarized in Table 2, GBM and glioblastoma stem-like cells (GSLCs) represent the most extensively investigated models [45,47]. In GBM, HK2 overexpression consistently correlates with increased glycolytic flux, enhanced proliferation, migration, and resistance to standard therapies. In contrast, in GSLCs, HK2 appears to play a central role in maintaining stemness and self-renewal capacity [45]. For example, Zhao et al. demonstrated that modulation of the miR-143/HK2 axis significantly reduced glycolysis and promoted differentiation in stem-like cell populations, highlighting the importance of HK2 in the maintenance of therapy-resistant tumor subclones [47].

The integration of HK2 expression within the context of glioma molecular heterogeneity highlights that it represents not only a marker of glycolytic activity but also a major determinant of tumor phenotype and clinical evolution [53]. In particular, the relationship between HK2 expression and IDH1 mutational status outlines the existence of distinct metabolic subtypes with relevant prognostic and therapeutic implications. IDH1-mutant tumors are characterized by an altered metabolism dominated by the production of 2-HG, which induces widespread epigenetic changes and reduces dependence on aerobic glycolysis. In this context, HK2 expression is generally decreased, and tumor cells exhibit a more pronounced metabolic orientation toward oxidative phosphorylation [53,54].

In contrast, IDH1 wild-type (GBM) show marked overexpression of HK2, associated with enhancement of the Warburg effect, increased glucose consumption, and elevated lactate production. This glycolytic phenotype correlates with accelerated proliferation, increased invasiveness, and poor prognosis [55,56]. Thus, correlating HK2 expression with IDH1 status allows the definition of two major metabolic subtypes: an aggressive glycolytic subtype characterized by high HK2 expression and IDH1 wild-type status, and a slower-evolving oxidative subtype characterized by low HK2 expression and IDH1 mutation. This classification has not only descriptive value but may also serve as a basis for clinically relevant stratification. From the perspective of patient stratification, these metabolic differences can significantly influence therapeutic decisions. Patients with tumors belonging to the glycolytic subtype may be more susceptible to therapies targeting glycolysis, including HK2 inhibitors or glucose analogs. However, the efficacy of such approaches is limited by the ability of tumor cells to activate compensatory metabolic pathways, such as fatty acid oxidation or glutaminolysis [57,58]. In this regard, combined therapeutic strategies targeting multiple metabolic pathways simultaneously are considered more promising. In contrast, IDH1-mutant tumors display distinct vulnerabilities, being more sensitive to therapies that exploit mitochondrial metabolism or the epigenetic effects of 2-HG accumulation. Specific IDH1 inhibitors, as well as epigenetic therapies, may have a more significant impact in this tumor subtype. Additionally, metabolic differences may influence the response to radiotherapy and chemotherapy by modulating oxidative stress levels and DNA repair capacity [54,59]. This distinction is particularly relevant when interpreting the studies included in Table 2, as several investigations specifically addressed GBM (IDH1 wild-type) models, whereas others included mixed-grade glioma cohorts. Therefore, discussing these tumor entities separately improves the biological interpretation of HK2-associated metabolic pathways and their potential translational relevance.

Another essential aspect is the interaction between glycolytic metabolism and the tumor microenvironment. HK2 overexpression contributes to increased lactate production, leading to extracellular acidification and suppression of the antitumor immune response. Lactate promotes macrophage polarization toward an immunosuppressive M2 phenotype and reduces the activity of cytotoxic T lymphocytes, thereby facilitating immune evasion [60]. Thus, HK2 plays a central role at the interface between tumor metabolism and immune regulation. Furthermore, HK2 is involved in regulating the transition toward more aggressive cellular phenotypes, including the mesenchymal subtype of GBM, characterized by increased invasiveness and therapeutic resistance. Interactions between HK2 and various regulatory molecules, such as microRNAs and lncRNAs, contribute to the remodeling of gene expression programs and increased tumor plasticity [61]. This plasticity represents a major factor in resistance to conventional therapies. Regarding treatment response, increased HK2 expression has been associated with resistance to temozolomide and radiotherapy. The underlying mechanisms include maintenance of energy production under stress conditions, reduced apoptosis, and mitochondrial protection [56,62]. Therefore, evaluating HK2 expression in correlation with IDH1 status may help identify patients at high risk of therapeutic resistance and guide treatment strategies. The integration of metabolic parameters, such as HK2 expression, into glioma molecular classification algorithms would allow a more refined characterization of tumors and open new perspectives for personalized therapies. Future approaches should aim not only to inhibit a single metabolic pathway but also to target the complex interaction networks that sustain tumor survival, including energy metabolism, intracellular signaling, and the immune microenvironment.

A less explored but biologically and clinically relevant aspect is the potential metabolic shift from glycolysis to OXPHOS in GBM cells and its implications for cellular differentiation. Several studies suggest that reduced reliance on glycolysis and increased mitochondrial flux may promote the activation of astrocyte-like differentiation programs [63,64]. This metabolic transition involves reprogramming of energy metabolism, reduced lactate accumulation, and increased mitochondrial ATP production, creating an intracellular environment more compatible with differentiated phenotypes. However, direct experimental evidence in GBM remains limited, and most data derive from *in vitro* models and studies analyzing differentiation marker expression under metabolic stress conditions. Therefore, conclusions regarding GBM cell differentiation through OXPHOS stimulation should be interpreted with caution and represent a promising area for future research [64,65].

Regarding the role of HK3 in cerebral glycolysis and GBM biology, this isoform has been less extensively studied compared to HK1 and HK2. HK3 is characterized by moderate glycolytic activity and predominantly cytosolic localization, unlike HK2, which associates with the outer mitochondrial membrane and integrates energy metabolism with survival signaling [66]. Several studies suggest that HK3 may contribute to maintaining energy homeostasis under stress conditions and modulate glycolytic flux in slow-cycling cellular subpopulations, frequently observed in GBM [67]. Compared to HK1 and HK2, HK3 appears to be more involved in regulating redox balance and reactive oxygen species production, providing additional protection against metabolic stress without generating the excess lactate characteristic of aggressive glycolytic phenotypes [68].

A comparative analysis of isoforms highlights that HK1 predominates in normal brain tissue and low-grade gliomas, ensuring basal glycolytic flux necessary for neuronal function, whereas HK2 is overexpressed in GBM, promoting accelerated glycolysis, the Warburg effect, and aggressive phenotypes [64,65]. In contrast, HK3 may act as a fine regulator of metabolism, maintaining a level of glycolysis compatible with the survival of slow-cycling tumor cells, thereby contributing to metabolic heterogeneity and therapeutic resistance [66,67,68]. In the clinical context, investigating HK3 expression in correlation with HK2 and IDH1 could enable the identification of tumor subpopulations with specific vulnerabilities that may be exploited through combined metabolic therapies targeting both glycolysis and OXPHOS, as well as compensatory pathways such as fatty acid oxidation and glutaminolysis [66,67]. An integrated understanding of the roles of HK1–3 in cerebral gliomas not only explains metabolic reprogramming and tumor plasticity but also facilitates the identification of more precise therapeutic targets. Future strategies should incorporate these insights to develop personalized therapies that target both aggressive glycolytic subtypes and slower-growing or predominantly mitochondrial subpopulations, thereby improving treatment efficacy and reducing tumor resistance [67,68].

## 4 Immunometabolic Mechanisms and the Interaction of HK with the Tumor Microenvironment

The metabolic reprogramming of malignant glial cells extends beyond the intensification of glycolysis and encompasses the complex interplay between energy metabolism and the tumor microenvironment. Over recent decades, studies have demonstrated that glycolytic enzymes—particularly the HKs isoforms HK1, HK2, and HK3—exert a dual role: they not only sustain the energetic and biosynthetic demands of tumor cells but also modulate the immune behavior of the surrounding microenvironment, influencing immune-cell infiltration, immune checkpoint expression, and the mesenchymal phenotype of the tumor [69]. While HK2 has traditionally been examined in the context of the Warburg effect and resistance to conventional therapies, recent interest has also focused on HK3, previously considered a minor isoform in neuronal metabolism but now recognized as a major participant in the immunometabolic processes of GBM. Unlike HK2, HK3 is predominantly expressed in infiltrating immune cells—especially tumor-associated macrophages (TAMs)—where it appears to promote polarization toward an immunosuppressive phenotype. These cells, through the release of anti-inflammatory cytokines and growth factors, establish a microenvironment that facilitates immune evasion and supports tumor progression [70,71].

### 4.1 Role of HK2 and Lactate in the Polarization of Tumor-Associated Macrophages (TAMs)

The high glycolytic metabolism of malignant glial tumor cells, characterized particularly by increased HK2 expression, leads to pyruvate accumulation [71]. Pyruvate is converted into lactate via LDH and subsequently exported from tumor cells through monocarboxylate transporters (MCTs) to prevent intracellular acidification and maintain metabolic homeostasis [72]. Once in the tumor microenvironment, lactate derived from HK2 activity acts as an immunometabolic mediator and influences TAM behavior [73].

Mechanistically, lactate affects TAMs through multiple intracellular signaling pathways. At the molecular level, lactate is transported into macrophages via the MCT1 transporter, which leads to the activation of transcription factors HIF-1α and HIF-2α, even in the absence of hypoxia, thereby stimulating the expression of genes associated with the immunosuppressive M2 phenotype, such as Arg1 and VEGF. The activation of these factors is linked to the induction of the mTOR pathway (mTORC1 and mTORC2 complexes) as well as STAT3 and AKT signaling, a molecular cascade that is critical for the metabolic and functional changes of TAMs toward an immunosuppressive and pro-tumoral profile [72]. In addition to classical signaling, lactate exerts effects through epigenetic mechanisms. Persistent lactate accumulation in the microenvironment is associated with an emerging post-translational modification: histone lactylation. This epigenetic modification remodels chromatin and allows increased expression of M2-associated genes, including Arg1 and others involved in tissue repair, regeneration, and inflammation suppression. Lactate-induced histone lactylation thus contributes to the long-term maintenance and stabilization of the immunosuppressive TAM phenotype, complementing classical metabolic signaling and making the M1 → M2 transition more robust in the presence of tumor-derived lactate [73].

The ERK/STAT3 signaling pathway is also activated by lactate, contributing to the expression of pro-tumoral factors and the functional conversion of TAMs. Lactate further enhances the expression of lactate-sensing receptors, such as GPR132 and GPR65, which modulate downstream macrophage responses and facilitate the secretion of immunosuppressive cytokines and angiogenic molecules [71]. Through activation of these pathways, TAMs lose their pro-inflammatory capacity and acquire a tumor-supportive profile by producing IL-10, TGF-β, and angiogenic factors such as VEGF and CCL17, collectively reducing the infiltration and cytotoxic efficacy of T cells (CTLs) and natural killer (NK) cells in the tumor [73].

Together, these mechanisms—increased glycolytic activity mediated by HK2, signaling through HIF-1α, STAT3, mTOR, and GPR pathways, epigenetic modifications via lactylation, and metabolic adaptations toward OXPHOS—highlight the central role of HK2 and tumor-derived lactate as immunometabolic mediators in tumor–immune system interactions [71,72]. By generating lactate, HK2 indirectly promotes TAM polarization toward an immunosuppressive M2 phenotype, thereby consolidating a pro-tumoral microenvironment, promoting angiogenesis and tumor growth, and limiting the effectiveness of antibody-based therapies and immunotherapies through immune evasion [72]. These findings suggest that therapeutic strategies targeting HK2 and lactate metabolism—including HK2 inhibition, reduction of lactate production, blockade of lactate transporters, or interference with lactate-sensitive signaling—may reprogram TAMs toward a more pro-inflammatory (M1) phenotype and improve responses to anticancer treatments, representing a promising avenue in combination cancer therapy [71].

### 4.2 The Involvement of HK2 and HK3 in Antitumor Immune Suppression and Remodeling of the Glial Microenvironment

Moreover, increased expression of HK2 and HK3 correlates with elevated levels of inhibitory immune markers such as PD-1, PD-L1, and CTLA-4, suggesting a direct link between activation of glycolytic pathways and suppression of antitumor immunity [23,45]. The underlying mechanisms involve not only transcriptional regulation but also intricate post-translational interactions through which HKs affect the stability of NF-κB pathway proteins and modulate gene expression related to antigen presentation [74,75].

The metabolic conditions of the microenvironment—particularly high glucose availability and hypoxia—further potentiate these immunosuppressive mechanisms. It has been shown that glucose abundance directly enhances PD-L1 expression in tumor cells via HK2. Under high-glucose conditions, HK2 dissociates from the mitochondrial membrane and interacts with IκBα, resulting in NF-κB activation and subsequent PD-L1 overexpression. This molecular shift enables tumor cells to evade cytotoxic T-cell attack and maintain a state of local immune tolerance. In GBM, HK3 expression is associated not only with poor prognosis but also with resistance to immunotherapy and chemotherapy. Recent transcriptomic analyses reveal that elevated HK3 levels correlate with activation of chemokine signaling pathways, recruitment of immunosuppressive immune subsets, and increased TIDE scores—indicative of heightened immune-escape capacity. Conversely, low HK3 expression corresponds to improved sensitivity to immunotherapy, suggesting that targeting HK3 could enhance the efficacy of immune-modulating treatments [71,74]. The close association of HK3 expression with M2 macrophage infiltration and malignant features of glial tumors suggests that this isoenzyme reshapes the immune microenvironment. Elevated HK3 levels consistently associate with recruitment of immunosuppressive immune cells, including M2-polarized TAMs, and with malignant progression, supporting the hypothesis that HK3 actively contributes to maintaining a pro-tumoral microenvironment [76,77]. Moreover, evidence indicates that HK3 expressed in TAMs can modulate their immunological functions through mTOR-dependent mechanisms, influencing antigen presentation and inhibiting cytotoxic T cell responses. This shows that HK3 can act both in tumor cells and in stromal cells, contributing to metabolic and functional coordination between tumor cells and macrophages, thereby extending the classical concept of “correlation” toward an active role in immunometabolic interactions [78].

Experimental studies also suggest that HK3 is not limited to correlation with M2 infiltration but may coordinate reciprocal responses between tumor and TAMs, promoting immunosuppressive polarization and supporting the M2 phenotype by modulating anti-inflammatory cytokines and arginine metabolism-associated enzymes. This duality of expression in tumor cells and macrophages indicates a bidirectional function, where HK3 not only reflects macrophage infiltration but also directly contributes to establishing an immunosuppressive microenvironment [79,80]. Correlations between HK3 and immune checkpoint marker presence suggest that this isoenzyme may also serve as a biomarker of the immune microenvironment, with prognostic and therapeutic potential. Interventions targeting HK3 could not only reduce TAM recruitment and immunosuppressive polarization but also restore antitumor immune responses and sensitivity to immunotherapy. These data also raise the question of whether HK3 is merely an effect of infiltration or an active driver of tumor–stroma interactions, justifying further mechanistic studies to clarify its functional role. Nevertheless, it should be emphasized that most available data remain correlational, and direct evidence confirming a causal role of HK3 in remodeling the immune microenvironment is still limited, requiring additional experimental validation [76].

Beyond their immunological implications, HK2 and HK3 also influence the genomic and phenotypic dynamics of glial tumors by contributing to extracellular matrix remodeling and regulation of angiogenesis. Their overexpression appears to promote mesenchymal transition, a process linked to increased aggressiveness, invasiveness, and treatment resistance. These findings underscore that HK2 and HK3 function not merely as metabolic enzymes but as key regulators of integrated tumor behavior at the interface of metabolism and immunity. Consequently, literature suggests that targeting HK2 and HK3 may provide multiple therapeutic advantages: decreasing glycolytic activity, restoring sensitivity to conventional treatments, and enhancing antitumor immunity. Notably, combining HK2 inhibitors with anti-PD-1 or anti-PD-L1 antibodies has shown promising results in experimental models, representing an emerging direction in personalized GBM therapy [70]. The following table (Table 3) summarizes the main experimental and bioinformatic studies investigating the roles of HK2 and HK3 in gliomas, highlighting their associations with the immune microenvironment, the relevant molecular mechanisms, and therapeutic implications. These data reinforce the concept of “tumor immunometabolism”, emphasizing the critical interplay between energy metabolism and immune responses in shaping prognosis and clinical evolution in GBM patients.

**Table 3:** Immunometabolic mechanisms and the interaction of HKs with the tumor microenvironment.

Study	Glial Entity/Tumor Type	Model	Mechanism/Target	Effect on Tumor	Therapeutic Potential	Future Directions
Liang et al. [76] *In silico*/Tissue analysis	GBM	Bulk RNA-seq (TCGA, CGGA validation), single-cell sequencing (CGGA), CCLE/BioGPS datasets, human GBM tissue IHC	HK3 → tumor-associated macrophages (CD163^+^) → immune microenvironment remodeling (TME); regulation of cancer immunity cycle (ssGSEA-based evaluation)	↑ macrophage infiltration, ↑ immunosuppression TME, impaired anti-tumor immune response, tumor progression	Target HK3 to modulate TME	Study HK3 molecular mechanisms and immunotherapy impact
Huang et al. [81] *In silico*/Database analysis	Glioma (GBM and other glioma histologies) + multiple malignant tumors	*In silico* analyses: ONCOMINE, GEPIA, TIMER2.0, CGGA (mRNA-seq, methylation, WES), UCSC Xena cohort (*n* ≈ 669 glioma patients), TCGA datasets	HK2 (hexokinase 2) → tumor metabolic reprogramming + association with immune infiltration and immune checkpoint activity	↑ HK2 expression in gliomas and multiple cancers vs. normal tissue; associated with higher tumor grade, immune infiltration, immune checkpoint activation, and poor prognosis	Target HK2 pathways to enhance therapy.	Explore HK2’s role in immune modulation
Yang et al. [70] *In silico*/Tissue analysis	GBM + pancancer cohort + glioma cell lines (U87, A172, U373, SVGp12)	Bulk RNA-seq (TCGA/CGGA), GTEx normal brain cortex (*n* = 207), single-cell datasets (TISCH), immune deconvolution (TIMER2.0, GSVA), drug response modeling (OncoPredict/Genomics of Drug Sensitivity in Cancer), *in vitro* qRT-PCR validation	HK3 → immune microenvironment regulation (T-cell dysfunction, macrophage infiltration, immune checkpoint pathways: PD-1/PD-L1/CTLA-4)	↑ Immune evasion, ↑ TIDE score (resistance to immunotherapy), poor survival; association with immunosuppressive microenvironment and therapy resistance	Target HK3 to improve immunotherapy response	Evaluate HK3 inhibitors and immune regulation

Note: ↑, increase/upregulation; →, leads to/results in; +, positive marker/presence of a marker; GBM, glioblastoma; TME, tumor microenvironment; IHC, immunohistochemistry; RNA-seq, RNA sequencing; mRNA-seq, messenger RNA sequencing; WES, whole-exome sequencing; qRT-PCR, quantitative reverse transcription polymerase chain reaction; ssGSEA, single-sample gene set enrichment analysis; GSVA, gene set variation analysis; GTEx, Genotype-Tissue Expression; TCGA, The Cancer Genome Atlas; CGGA, Chinese Glioma Genome Atlas; CCLE, Cancer Cell Line Encyclopedia; TIDE, Tumor Immune Dysfunction and Exclusion.

Liang et al. [76] and Yang et al. [70] demonstrated that HK3 is overexpressed in gliomas and is associated with increased macrophage infiltration and the establishment of an immunosuppressive tumor microenvironment (TME). Moreover, HK3 expression correlates with key regulators of immune response, including PD-1, PD-L1, and CTLA-4, suggesting that this enzyme may facilitate immune evasion and contribute to treatment resistance. In addition, TIDE analysis indicates that tumors with low HK3 expression may respond more favorably to immunotherapy, highlighting the clinical potential of HK3 inhibition as a complementary therapeutic strategy. These findings support the concept that tumor metabolism and immune regulation are tightly interconnected and that therapies targeting a single metabolic node may be insufficient without the simultaneous modulation of TME components.

Huang et al. [81] provide complementary evidence, showing that HK2 overexpression correlates with increased expression of immune checkpoint molecules and with poor prognosis, further emphasizing the link between glycolytic metabolism and immune regulation. These observations suggest that HKs function not only as metabolic enzymes but also as signaling mediators that regulate immune activity within the tumor microenvironment. In this context, HK3 and HK2 can be regarded as critical nodes linking tumor metabolism to immune evasion and therapeutic resistance. More broadly, the integration of these findings highlights several strategic directions for future research and the development of personalized therapies: (i) direct inhibition of HK3 and HK2 to reduce glycolysis and sensitize tumors to immunotherapy; (ii) combining metabolic therapies with immune checkpoint blockade to counteract the immunosuppressive effects of the TME; (iii) identifying patients with high HK3/HK2 expression and an immunosuppressive TME as potential candidates for combination therapies; and (iv) exploring the role of other HK isoforms and upstream epigenetic and metabolic networks that influence both tumor progression and immune responses.

Overall, the evidence presented in this study positions HK2 and HK3 not as isolated metabolic enzymes, but as central integrators within a broader immunometabolic network that governs glioma progression. Beyond their classical role in regulating glycolytic flux, these isoforms emerge as key nodes linking metabolic reprogramming to immune checkpoint activation, macrophage polarization, extracellular matrix remodeling, and angiogenic signaling, thereby placing glioblastoma within a systemic framework of tumor–stromal co-evolution [69,70,71]. In this context, glycolysis is no longer merely an energy-producing pathway, but a regulatory platform that coordinates transcriptional, epigenetic, and signaling circuits across both tumor and immune compartments [72,73]. Importantly, multi-omics studies and single-cell analyses extend this model, demonstrating that metabolic heterogeneity in glioma mirrors immune heterogeneity, with HK2- and HK3-high states consistently correlating with myeloid immunosuppressive infiltration, T-cell dysfunction, and mesenchymal transcriptional programs [70,76,81]. This integrated perspective aligns with emerging concepts in cancer immunometabolism, in which metabolic enzymes act as functional mediators of immune evasion rather than passive metabolic effectors [69,71]. Consequently, HK2 and HK3 represent convergence points between oncogenic signaling pathways, including NF-κB, STAT3, and HIF-1α, and microenvironmental adaptation, reinforcing the concept that glioma progression is driven by tightly coupled metabolic–immune feedback loops rather than independent cellular processes [72,74,80]. These data further support the development of combinatorial therapeutic strategies simultaneously targeting metabolic rewiring and immune checkpoint pathways, aiming to disrupt the interdependence between tumor glycolysis and immunosuppressive microenvironmental conditioning [70,81].

## 5 HKs as a Therapeutic Target

### 5.1 Metabolic Therapies and Their Limitations

One of the most extensively studied strategies involves the use of 2-DG and its halogenated analogs (2-fluoro-D-glucose, 2,2-difluoroglucose, 2-chloro-D-glucose, or 2-bromo-D-glucose), Fig. 2. These molecules mimic the structure of glucose and bind to HK2, where they are phosphorylated but cannot proceed through normal metabolic cycles, leading to glycolysis inhibition and reduced ATP production. Consequently, tumor cells experience an energy deficit, which can trigger apoptosis or increase sensitivity to chemotherapy and radiotherapy. Halogenated analogs demonstrate greater stability and improved cellular uptake compared to 2-DG, and preclinical studies suggest they remain effective even under hypoxic conditions, which are common in the tumor microenvironment [51]. In parallel, specific small-molecule HK2 inhibitors have been developed, directly blocking enzymatic activity without relying on glucose mimetics. These compounds reduce cell proliferation, induce metabolic stress, and can decrease resistance to standard therapies. Their effects have been demonstrated both *in vitro* and in animal models, and research continues to identify compounds with optimal pharmacokinetics and low toxicity [82].

A complementary and innovative approach involves combining HK2 inhibition with immunotherapy. Preclinical studies combining HK2 inhibitors with anti-PD-1 antibodies have shown increased CD8^+^ T-cell infiltration into tumors and reduced immune evasion [21,48]. This suggests that targeting glycolytic metabolism can remodel the tumor microenvironment, enhancing immune responses and the therapeutic potential of existing immunotherapies. Epigenetic modulation of HK2 expression represents another promising strategy. HOTAIR inhibitors, miR-125 modulators, or miR-542-3p agonists reduce HK2 mRNA or protein levels, thereby decreasing glycolysis and tumor proliferation while increasing sensitivity to TMZ. Similarly, blocking the CASC9/IGF2BP2 complex reduces HK2 mRNA stability and limits aerobic glycolysis, representing an innovative approach to regulating tumor metabolism through post-transcriptional mechanisms, Fig. 2 [50,71].

**Figure 2:**
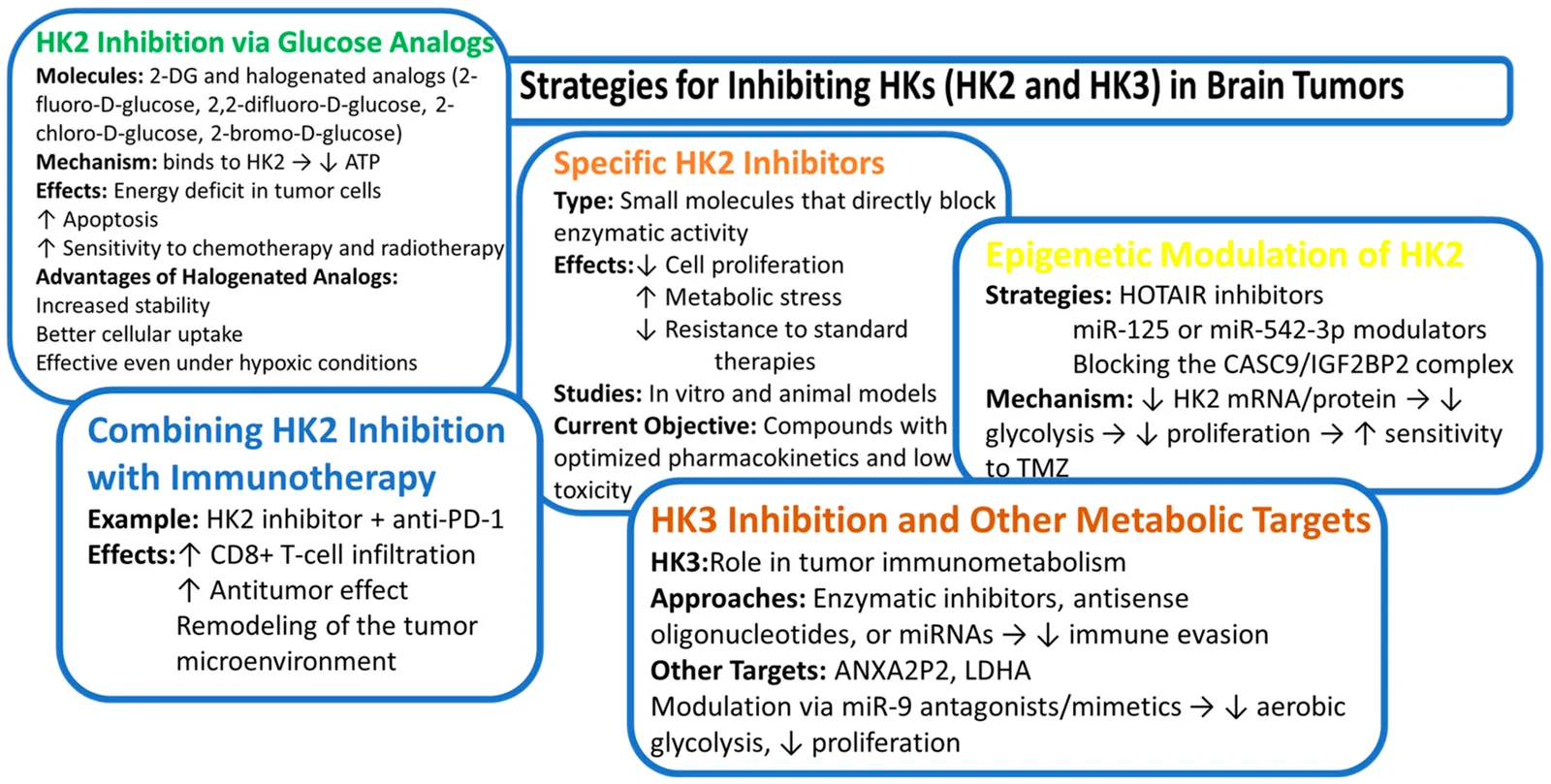
Schematic overview of regulatory pathways and therapeutic interventions targeting HK2 and HK3 in gliomas. HK2 is modulated by miR-21 and miRAIR, with potential inhibition via 2-DG analogs and ANXA2P3, while HK3 influences PD-1/PD-L1 signaling and can be targeted by miR-542-3p or specific HK3 inhibitors. ↑ increase; ↓ decrease; → leads to/results in. HK2/HK3, hexokinase 2/hexokinase 3; ATP, adenosine triphosphate; PD-1, programmed cell death protein 1; HOTAIR, HOX Transcript Antisense Intergenic RNA; miR/miRNA, microRNA; CASC, cancer-associated susceptibility candidate; IGF2BP2, insulin-like growth factor 2 mRNA-binding protein 2; TMZ, temozolomide; CD8^+^ T cell, CD8-positive cytotoxic T lymphocyte; LDHA, lactate dehydrogenase A; ANXA2P2, annexin A2 pseudogene 2. Created with BioRender.com (BioRender Inc., version 2.1, 2022, https://www.biorender.com).

HK3, less extensively studied, is involved in regulating tumor immunometabolism and can influence the activation of immunosuppressive macrophages [43]. Specific enzymatic inhibitors of HK3, as well as antisense or miRNA-based approaches, may reduce immune evasion and enhance antitumor immune responses, and are currently under exploratory investigation. Additionally, other related metabolic targets, such as ANXA2P2 or LDHA, can be modulated using miR-9 antagonists or mimetics, reducing aerobic glycolysis and cell proliferation. These findings highlight the importance of a combined therapeutic approach that simultaneously targets energy metabolism and the immune microenvironment, opening avenues for integrated treatment strategies [70,71].

However, despite the promising results obtained in preclinical studies, the clinical translation of strategies based on 2-DG and its halogenated analogs has faced significant limitations. Phase II and III clinical trials have shown modest efficacy, accompanied by a range of adverse effects that limit widespread use. Major obstacles include systemic toxicity, particularly hypoglycemia, fatigue, and effects on normal tissues dependent on glycolysis [83]. These effects can be explained by the lack of specificity of 2-DG, which does not distinguish between tumor cells and normal cells with active glycolytic metabolism [40,84]. Another major mechanism contributing to the failure of these strategies is the metabolic plasticity of tumor cells. Under glycolytic inhibition, neoplastic cells can activate compensatory metabolic pathways to maintain survival and energy production. Studies have shown that glycolysis inhibition increases flux through β-oxidation, facilitating ATP and NADH production in mitochondria [58,85]. This metabolic reprogramming is mediated by transcription factors such as PPARα and the coactivator PGC-1α, which stimulate the expression of FAO-related enzymes [86]. In GBM, this metabolic adaptation has been associated with more aggressive phenotypes and increased therapy resistance [87]. Moreover, tumor cells can increase their dependence on glutamine metabolism (glutaminolysis), using it as an alternative carbon and nitrogen source for the tricarboxylic acid (TCA) cycle and macromolecule biosynthesis. Activation of glutaminase and glutamine transporters allows maintenance of energy homeostasis even under glycolytic inhibition [88]. Thus, monotherapy exclusively targeting glycolysis proves insufficient, as it is rapidly countered by tumor metabolic flexibility [57,89].

Another critical aspect is intratumoral heterogeneity, a defining feature of GBM. Distinct cellular subpopulations may exhibit different metabolic phenotypes, with some being predominantly glycolytic and others oxidative [87]. In this context, inhibition of a single metabolic pathway fails to uniformly affect all tumor compartments, favoring the selection of resistant clones. Hypoxic conditions within the tumor microenvironment further contribute to this heterogeneity by stabilizing HIF-1α, promoting anaerobic glycolysis, and inducing alternative adaptive mechanisms [86]. Pharmacokinetic barriers further compound these limitations. Inadequate penetration of the blood–brain barrier reduces effective 2-DG concentrations within the tumor, limiting therapeutic impact. Additionally, rapid clearance and variable bioavailability contribute to the inconsistent clinical responses observed [83].

In this context, it becomes clear that effective therapeutic strategies must adopt a combinatorial approach, targeting multiple metabolic pathways simultaneously. For example, combining glycolytic inhibitors with fatty acid oxidation inhibitors (such as etomoxir) or glutaminolysis inhibitors (such as GLS inhibitors) has shown promising results in preclinical models, suggesting potential therapeutic synergy [85,88]. Integration of these strategies with standard therapies or immunotherapy may further enhance antitumor effects by limiting the metabolic adaptability of tumor cells [87]. Thus, although targeting glycolysis through 2-DG and its analogs represents an attractive theoretical strategy, clinical efficacy is limited by toxicity, lack of specificity, and, most importantly, the remarkable ability of tumor cells to reprogram their metabolism [87,89].

### 5.2 Epigenetic Strategies as Therapeutic Alternatives

Given the significant limitations of conventional metabolic therapies, there is a pressing need to explore alternative strategies capable of modulating tumor metabolism in a more complex and adaptive manner. In this context, the epigenetic regulation of genes involved in tumor metabolism has emerged as a promising avenue, although its therapeutic potential remains insufficiently explored. Regarding glycolytic metabolism, HKs, particularly HK2 and HK3, are integrated into a complex epigenetic regulatory network that includes microRNAs (miRNAs), long non-coding RNAs (lncRNAs), chromatin modifications, and interactions with RNA-binding proteins [71]. These mechanisms not only modulate HK expression but also contribute to tumor heterogeneity, metabolic adaptation, and treatment resistance, as summarized in Table 2. HK3, overexpressed in gliomas, promotes macrophage infiltration, contributing to an immunosuppressive tumor microenvironment and malignant progression, whereas HK2 regulates PD-L1 expression via interactions with IκBα and NF-κB, modulating CD8^+^ T-cell infiltration and promoting immune evasion. These mechanisms suggest that HK2 inhibition combined with immune checkpoint blockade could enhance therapeutic efficacy [76,81].

The first level of epigenetic regulation involves miRNAs, which act post-transcriptionally by degrading mRNA or inhibiting translation. For instance, miR-218, miR-125, and miR-1271-5p have been described as indirect inhibitors of HK2, either by repressing upstream regulators (such as Bmi1) or through direct interactions with HK2 mRNA [42,44]. These data are mainly derived from *in vitro* studies and animal models, indicating a promising therapeutic potential, but still insufficiently validated clinically. Similarly, miR-9 influences glycolytic metabolism via LDHA, highlighting that epigenetic networks do not act in isolation but coordinate the overall tumor metabolic flux [43,48].

LncRNAs represent a second major level of epigenetic regulation, often acting as “sponges” for miRNAs or as interaction platforms for protein complexes. Relevant examples include HOTAIR, ZFAS1, and CASC9, which modulate HK2 expression through indirect mechanisms affecting mRNA stability or the availability of inhibitory miRNAs. For instance, the CASC9/IGF2BP2/HK2 axis demonstrates a post-transcriptional mechanism whereby HK2 mRNA stabilization supports aerobic glycolysis and tumor proliferation [42,48]. However, most of these data come from experimental studies, and their clinical relevance is still under investigation. Additionally, the expression of these lncRNAs can vary significantly between molecular glioma subtypes, highlighting the need for patient stratification based on epigenetic profiles [50].

An insufficiently explored but essential aspect involves epigenetic chromatin modifications, including DNA methylation and histone modifications. Although indirect evidence suggests that promoters of glycolysis-related genes can be regulated via methylation or acetylation, data specific to HK2 and HK3 in glioma are limited. In this context, integrating histone deacetylase (HDAC) inhibitors or methyltransferase inhibitors could represent a promising therapeutic direction, especially in combination with metabolic therapies. However, the lack of specific studies correlating these modifications with HK expression limits the immediate applicability of such strategies. Therapeutically, targeting epigenetic regulators offers significant theoretical advantages compared with direct inhibition of metabolic enzymes. Such approaches may be more selective, modulating tumor-specific networks. Moreover, they can counteract tumor heterogeneity by simultaneously affecting multiple signaling pathways. Nevertheless, substantial challenges remain. Efficient delivery of miRNA- or antisense-based therapies is limited by biological barriers, including the blood–brain barrier. Additionally, off-target effects and molecular instability constitute major obstacles for clinical translation. Another important consideration is the current level of evidence: most data on epigenetic regulators come from *in vitro* studies or animal models, with a limited number of *in silico* analyses, underscoring the need to validate these mechanisms in patient cohorts. Furthermore, differences among glioma types (e.g., GBM versus lower-grade astrocytoma) are rarely systematically addressed, although these entities exhibit distinct epigenetic profiles [45,51].

The integration of epigenetic regulators into therapeutic strategies, therefore, requires a multidimensional approach. First, epigenetic data should be correlated with molecular glioma subtypes to identify predictive biomarkers of treatment response [21,44]. Second, combining epigenetic therapies with metabolic inhibitors or immunotherapy may enhance antitumor effects by simultaneously blocking multiple adaptive mechanisms. Third, the development of efficient delivery systems, such as nanoparticles or viral vectors, could overcome current limitations in bioavailability.

## 6 Conclusions

Metabolic intervention in glioma through inhibition of HK2 and HK3 represents a promising therapeutic strategy, exerting direct effects on cell proliferation and sensitivity to conventional treatments. Modulation of microRNAs involved in regulating these enzymes, provides an additional approach to reduce glycolytic activity and diminish tumor resistance to chemotherapy and radiotherapy. This dual effect, impacting both metabolism and cell survival, highlights the central role of HK2 and HK3 in glioma biology and supports further research in this field.

Combining metabolic therapy with immunotherapy, particularly through PD-1/PD-L1 inhibitors, represents an emerging direction with significant clinical potential. Overall, targeting HK2 and HK3 may exert dual antitumor effects by reducing tumor cell proliferation and modulating the immune microenvironment, thereby potentially enhancing CD8^+^ T-cell infiltration and antitumor immune responses. This combined strategy supports the development of synergistic therapeutic approaches that could overcome the limitations of standard treatments and improve clinical outcomes in glioma patients.

Halogenated analogs of 2-DG currently constitute the most advanced class of compounds targeting glycolysis, being extensively tested in preclinical models due to their increased stability and efficacy, even under hypoxic conditions. In parallel, inhibitors of the HOTAIR/miR-125, CASC9/IGF2BP2, and ANXA2P2/miR-9 axes are still in the preclinical stage but show potential for synergistic combinations with TMZ or immunotherapy, opening new avenues for personalized treatments.

Recent data emphasize the importance of an integrated approach that simultaneously targets tumor metabolism, metabolic adaptation, and the immune microenvironment. Tumor metabolic plasticity, intratumoral heterogeneity, and pharmacokinetic barriers can limit the efficacy of monotherapies, making multi-target combination strategies essential. Moreover, patient stratification based on molecular and epigenetic biomarkers may optimize treatment response and guide the development of precision oncology approaches.

Overall, current data suggest that an integrated approach targeting glycolytic metabolism and the immune microenvironment could represent a viable and innovative strategy for glioma treatment. Continued preclinical studies and transition to clinical trials will be essential for validating these therapies and optimizing therapeutic combinations, aiming to enhance efficacy and reduce tumor resistance. This direction represents an important step toward the development of personalized therapeutic strategies that simultaneously target tumor proliferation and immune evasion.

## Data Availability

Not applicable.
